# Exploring Cell Wall Composition and Modifications During the Development of the Gynoecium Medial Domain in *Arabidopsis*

**DOI:** 10.3389/fpls.2018.00454

**Published:** 2018-04-12

**Authors:** Humberto Herrera-Ubaldo, Stefan de Folter

**Affiliations:** Unidad de Genómica Avanzada, Laboratorio Nacional de Genómica para la Biodiversidad, Centro de Investigación y de Estudios Avanzados del Instituto Politécnico Nacional, Irapuato, Mexico

**Keywords:** cell wall, gynoecium development, microscope imaging, polysaccharides, fertilization, plant reproduction, *Arabidopsis thaliana*, flower development

## Abstract

In *Arabidopsis*, the gynoecium, the inner whorl of the flower, is the female reproductive part. Many tissues important for fertilization such as the stigma, style, transmitting tract, placenta, ovules, and septum, comprising the medial domain, arise from the carpel margin meristem. During gynoecium development, septum fusion occurs and tissues form continuously to prepare for a successful pollination and fertilization. During gynoecium development, cell wall modifications take place and one of the most important is the formation of the transmitting tract, having a great impact on reproductive competence because it facilitates pollen tube growth and movement through the ovary. In this study, using a combination of classical staining methods, fluorescent dyes, and indirect immunolocalization, we analyzed cell wall composition and modifications accompanying medial domain formation during gynoecium development. We detected coordinated changes in polysaccharide distribution through time, cell wall modifications preceding the formation of the transmitting tract, mucosubstances increase during transmitting tract formation, and a decrease of mannan distribution. Furthermore, we also detected changes in lipid distribution during septum fusion. Proper cell wall composition and modifications are important for postgenital fusion of the carpel (septum fusion) and transmitting tract formation, because these tissues affect plant reproductive competence.

## Introduction

**FIGURE 1:**
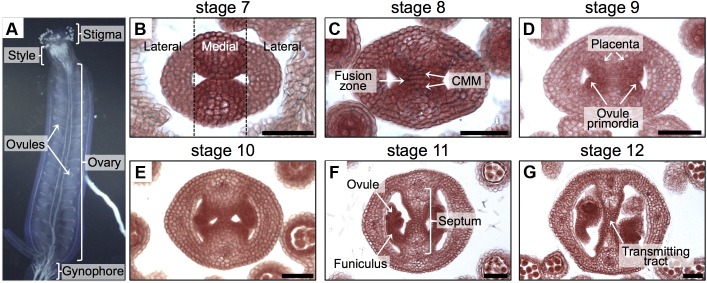
Overview of the gynoecium and its development in *Arabidopsis*. **(A)** Lateral view of the *Arabidopsis gynoecium*. **(B–G)** Transverse sections of *Arabidopsis* gynoecia stained with Neutral red at stages 7 **(B)**, 8 **(C)**, 9 **(D)**, 10 **(E)**, 11 **(F)**, and 12 **(G)**. Annotations in **(A)** mark tissues of a mature gynoecium; the dotted lines in **(B)** separate the lateral domains from the medial domain; annotations in **(B–G)** mark the regions or tissues in developing gynoecia. Scale bars represent 50 μm **(B–G)**.

The *Arabidopsis thaliana* gynoecium is the female part of the flower and contains the female reproductive tissues and organs important for pollination, fertilization, and finally seed formation. At the apical part, we can find the stigma that captures pollen grains and helps them germinate, then the pollen tubes grow through the style and septum via the transmitting tract in order to reach the ovules in the ovary (**Figure 1A**), so that fertilization can occur and seed development starts (Smyth et al., 1990; Bowman et al., 1999; Alvarez-Buylla et al., 2010; Reyes-Olalde et al., 2013).

Most of the tissues and processes affecting reproductive competence belong to the medial domain of the gynoecium (**Figure 1**). In *Arabidopsis*, the gynoecium is formed by the fusion of two carpels, which fuse vertically at their margins and thereby giving rise to the carpel margin meristem (CMM; **Figure 1B**; Reyes-Olalde et al., 2013). This domain is very dynamic; many tissues are continuously arising in the time frame of a few days, like the septum that is formed by the fusion of the two septa primordia (**Figures 1C,D**), placenta tissue and ovule primordia arise (**Figures 1C,D**). Furthermore, another important event is the formation of the transmitting tract (**Figures 1E–G**), allowing pollen tubes to reach the ovules and therefore fertilization efficiency. Transmitting tract formation relies on programmed cell death (Crawford et al., 2007; Crawford and Yanofsky, 2008) and on the excretion of a special extracellular matrix (ECM), a mixture of glycoproteins, glycolipids, and polysaccharides that helps pollen tubes in their journey through the pistil (Lennon et al., 1998).

Furthermore, for dehiscent fruits, like in *Arabidopsis*, lignification of the cell wall of specific cells has to occur so that the valves can detach when the fruit is dry and the seed can disperse (Ballester and Ferrandiz, 2017). A similar process occurs in the funiculus, the tissue connecting placenta and seed, necessary for seed abscission (Balanzà et al., 2016).

During several of the previously mentioned events, modifications at the cellular level of the cell walls have to take place. Gynoecium medial domain development has been commonly studied using histochemical methods such as Alcian blue staining for the visualization of the transmitting tract (e.g., Alvarez and Smyth, 2002; Zúñiga-Mayo et al., 2012) or Neutral red and Toluidine blue to observe cell morphology. However, there are many other dyes or fluorescent dyes or molecules that are not well explored in a more systematic way to observe them during gynoecium development.

In this work, we explored and monitored the composition of some modifications of the cell wall during gynoecium medial domain development. We used some of the classical staining protocols, several fluorescent dyes, and also immunofluorescence labeling. Moreover, detailed protocols are provided with technical tips how to perform the staining and immunolocalization procedures. With all this, we tracked cellulose, mannan, glycoproteins (mucosubstances), lignin deposition, and neutral lipid distribution during gynoecium development.

## Materials and Methods

### Sample Preparation

*Arabidopsis thaliana* Col-0 and *ntt-3* (Marsch-Martinez et al., 2014) plants were grown in soil in a growth chamber at 22°C, under a 16 h light/8 h dark photoperiod. *Arabidopsis* inflorescences with flowers from stages 6 to 13 (Smyth et al., 1990) were collected in 50 mL falcon tubes containing 10 mL of fixation solution [3% paraformaldehyde in PBS for immunolabeling or FAE (5% formaldehyde, 10% acetic acid, 50% ethanol) for other stains]. Tubes were placed in a vacuum desiccator and vacuum was applied for 15 min. Afterward, samples were incubated at room temperature for 2 h. The tissue was dehydrated by passing through a series of ethanol solutions (20, 30, 50, 70, 85, and 100% ethanol); 1 h each, at room temperature.

### Tissue Embedding, Blocking, and Sectioning

Samples were embedded in acrylate according to manufacturer instructions; we used the reagent Technovit (Heraeus Kulzer, Germany). A block contained one or two inflorescences. Blocks were sectioned using a microtome; sections were 12–18 μm thick. The quality of the sections was checked by placing them on a glass slide with water, after that, 10–15 sections were transferred to one well (24-well plate) with 1 mL distilled water (free floating of tissue sections; Supplementary Figure 1).

### Bright Field Microscopy

Sections were transferred to glass slides, air dried, and used for the following staining procedures: Neutral red staining (Sigma-Aldrich; 0.5% dissolved in water) samples were stained for a few minutes, washed with water for 1 min, and air-dried (Zúñiga-Mayo et al., 2012).

Alcian blue 8GX staining (Sigma-Aldrich; 0.3% dissolved in water, then adjusted to pH 3.1 or 2.5 with acetic acid; or pH 1.0 with HCl), samples were stained for 10 min, washed with water for 1 min, and air-dried (Sessions and Zambryski, 1995; Zúñiga-Mayo et al., 2012).

Phloroglucinol staining (Sigma-Aldrich; 2% dissolved in 100% ethanol), samples were stained for 1 min, then washed with 50% HCl for 1 min, and observed immediately (Balanzà et al., 2016).

Toluidine blue staining (HYCEL, Zapopan, Mexico; 0.02% Toluidine blue solution in water), samples were stained for 3 min, washed with water for 1 min, and air-dried (Mitra and Loque, 2014).

Congo red staining (0.5% Congo red solution in water), samples were stained for 5 min, washed with water for 1 min, and air-dried (Mitra and Loque, 2014; Ribas-Agusti et al., 2014).

### Confocal and Fluorescence Microscopy

In general, floating sections were transferred to a well (24-well plate) containing the staining solution, washed with water, then transferred to glass slides and observed immediately. Fluoroshield (Sigma-Aldrich) was used as mounting medium.

For the acriflavine staining, 20 μL of stock solution (Acrivelt-Biomaa, Mexico; 0.11% solution dissolved in water) was added to 1 mL of water, sections were stained in this solution for 5 min, then washed with water, and mounted on a glass slide. Samples were observed in a confocal laser scanning-inverted microscope LSM 510 META (Carl Zeiss, Germany); for the detection of lignified tissue, excitation was done with a 488-nm laser line of an Argon laser and emission was filtered with a BP 500–520 nm filter; for the observation of non-lignified tissues, excitation was done with a 488 and 514 nm laser lines and emission was filtered with a LP 575 nm filter (Donaldson et al., 2001; Bonawitz et al., 2014). Samples were also observed with a Leica DM6000B using a N2.1 filter (excitation filter: 515–560 nm and detection filter LP 590 nm), with a UV lamp.

For the Calcofluor white staining, 100 μL of stock solution (FLUKA, Sigma-Aldrich; Calcofluor white M2R 1 g L^-1^, Evans blue 0.5 g L^-1^) was added to 1 mL of water, sections were stained in this solution for 5 min, washed with water, and mounted on a glass slide (Mitra and Loque, 2014; Herburger and Holzinger, 2016). Samples were observed with a Leica DM6000B using an A filter cube (excitation filter: 340–380 nm and detection filter LP 425 nm), with a UV lamp.

For the Nile red staining, 4 μL of stock solution (500 μg mL^-1^ dissolved in acetone) was added to 1 mL of 75% glycerol, some droplets were added on a glass slide containing air-dried sections, a coverslip was placed and observed immediately with a LSM 510 META confocal microscope; the fluorophore was excited with a 488 and 514 nm laser lines and emission was filtered with a BP 575–615 IR filter (Kuntam et al., 2015).

In the case of propidium iodide (PI) staining, 1 μL of stock solution (Sigma-Aldrich; 5 mg mL^-1^ dissolved in water) was added to 1 mL of water, sections were stained in this solution for 30 s, washed with water, and mounted on a glass slide. Samples were observed with a confocal laser scanning-inverted microscope LSM 510 META (Carl Zeiss, Germany), the fluorophore was excited with a 514 nm laser line, and emission was filtered with a LP 575 nm filter (Reyes-Olalde et al., 2015). The following objectives were used: EC Plan-Neofluar 20×/0.5 and EC Plan-Neofluar 40×/0.75 (Reyes-Olalde et al., 2015).

### Mannan Immunolabeling

We started with sections in water in the 24-well plate (Supplementary Figure 1). The distilled water was removed from the well with a pipette, followed by the addition of 2 mL of 1 M KOH and incubation for 1 h at room temperature; this treatment was performed to expose hidden mannans (Marcus et al., 2010). The KOH solution was removed and samples were washed three times with Buffer 1 (1% PBS, pH 7, 2% BSA); 1.5–2 mL of buffer was used for each wash step. We used LM21 as primary antibody (monoclonal, Rat IgM, Plant Probes, United Kingdom). For the hybridization with the primary antibody, hybridoma supernatant was diluted 1:50 with Buffer 1; incubation was done at 26°C for 16 h. Solution with primary antibody was removed and samples were washed three times with 2 mL of Buffer 1. For the hybridization with the secondary antibody, we used a 1:1000 dilution in Buffer 1; incubation was done at 26°C for 4 h. Solution with secondary antibody was removed and samples were washed three times with Buffer 1.

As negative controls, we treated the sections with cellulase and macerozyme (Yakult, Japan; 5 and 5 mg mL^-1^, respectively, dissolved in distilled water with pH 5.7), which are enzymes used in the first step of the preparation of protoplasts (Yoo et al., 2007). The treatment was done before the incubation with KOH, for a short time (15 min) followed by washing three times with distilled water. Alternatively, we processed samples without adding the primary antibody.

The sections were taken out of the Buffer 1 with the forceps and transferred to a microscope glass slide with glycerol 50%; the solution facilitated the complete expansion of the section (needles and forceps were used). The excess of glycerol was removed and a coverslip was placed. Samples were observed using a Confocal Laser Scanning Microscope (CLSM) and a fluorescence microscope, as reported (Reyes-Olalde et al., 2015). As secondary antibody we used the Goat Anti-Rat IgM mu chain (DyLight^®^ 488) from Abcam (Cambridge, United Kingdom). This fluorophore is excited with a 488 nm laser and the emission is at 518 nm. Therefore, to capture the fluorescent images, we used a confocal laser scanning-inverted microscope LSM 510 META (Carl Zeiss, Germany). DyLight^®^ 488 (Abcam, Cambridge, United Kingdom) was excited with a 488 nm laser line of an Argon laser and emission was filtered with a BP 500–520 nm filter. The following objectives were used: EC Plan-Neofluar 20×/0.5 and EC Plan-Neofluar 40×/0.75 (Reyes-Olalde et al., 2015). Samples were also observed with a Leica DM6000B using a GFP filter cube (excitation filter: 470/40 nm and detection filter BP 525/50 nm) with a UV lamp.

## Results

In this study, we analyzed cell wall composition and modifications that occur during medial domain development in the gynoecium of *Arabidopsis*. We used the following classical staining methods: Neutral red, Phloroglucinol, Toluidine blue, Alcian blue, and Congo red. Furthermore, we used the common fluorescent dye Calcofluor white, and, less frequently used in gynoecium studies, Acriflavine and Nile Red. Additionally, we set-up indirect immunolocalization of cell wall components in gynoecium cross-sections. In the section “Materials and Methods,” detailed information is provided how to perform and visualize all these techniques. With all this, we monitored the distribution of lignin, mucosubstances (glycoproteins), cellulose, mannans, and lipids during gynoecium development.

### Lignin Deposition During Gynoecium Development

**FIGURE 2:**
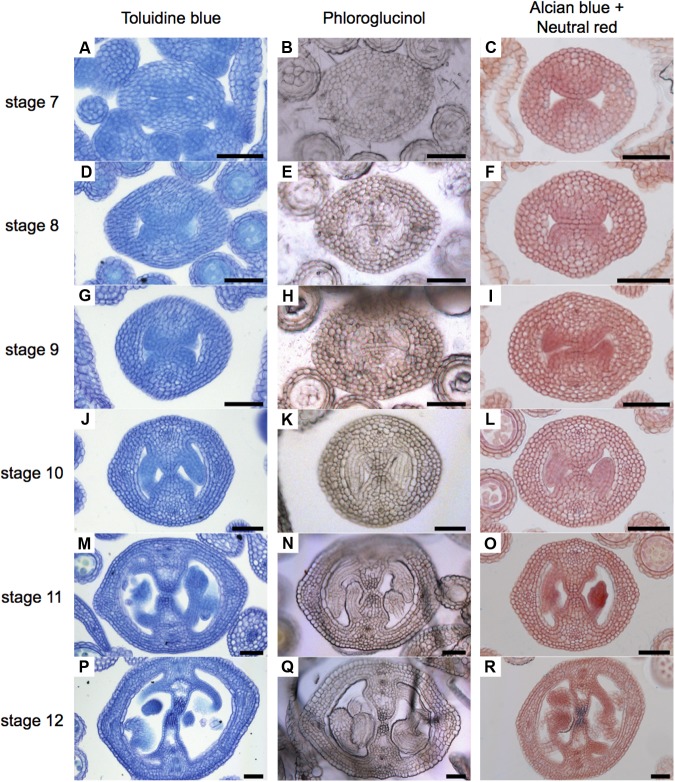
General overview of stained cell walls in transverse sections of *Arabidopsis* gynoecia. **(A–R)** Transverse sections of *Arabidopsis* gynoecia stained with Toluidine blue **(A,D,G,J,M,P)**, Phloroglucinol **(B,E,H,K,N,Q)**, or Neutral red and Alcian blue **(C,F,I,L,O,R)** at stages 7 **(A–C)**, 8 **(D–F)**, 9 **(G–I)**, 10 **(J–L)**, 11 **(M–O)**, and 12 **(P–R)**. Scale bars represent 50 μm **(A–R)**.

In order to obtain a general overview of the cell wall contents during gynoecium development, we first used Toluidine blue staining (**Figure 2**), a polychromatic stain, allowing the visualization of several compounds (e.g., nucleic acids stain blue and polysaccharides stain purple) in the same sample (O’brien et al., 1964). As expected, we detected bluish cell walls and some dark blue/purple staining. Notably, a dark blue color was detected in the region of the transmitting tract (**Figures 2J,M,P**). This pattern could be observed from stage 10 and followed the formation of the transmitting tract.

Lignin is a complex organic polymer and is important for cell walls, but can also be found in between cells and in the cell of all vascular plants (Vanholme et al., 2010). We monitored lignin deposition with Phloroglucinol staining (**Figure 2**), specific for lignin-associated hydroxycinnamaldehydes (Bonawitz et al., 2014). Lignin was detected in vascular tissues (**Figures 2N,Q**). Furthermore, we detected some signal at stage 8 in the medial region (**Figure 2E**), and this signal increased until stage 12, accompanying the formation of the transmitting tract (**Figures 2E,H,K,N,Q**). Finally, we focused specifically on the formation of the transmitting tract, important for pollen tube passage (Crawford and Yanofsky, 2008). For this, we used the common Alcian blue (pH 3.1) staining method, which allows the visualization of polysaccharide compounds in blue (Alvarez and Smyth, 2002; Crawford et al., 2007), and Neutral red as a counter stain (**Figure 2**). The first blue stains were detected at stage 9 (**Figure 2I**), this color was very light and only visible in a few cells, the color intensity increased through time until stage 12, when the transmitting tract is mature (**Figures 2L,O,R**).

**FIGURE 3:**
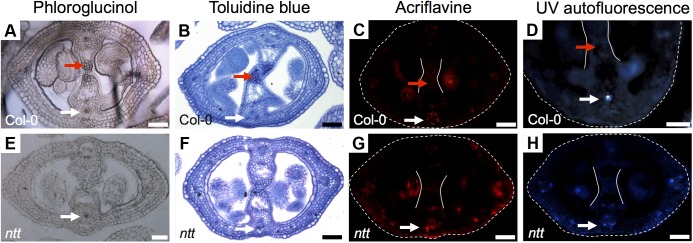
Detection of lignin *Arabidopsis* gynoecia. Phloroglucinol **(A,E)**, Toluidine blue **(B,F)** acriflavine **(C,G)** staining, and UV-autofluorescence **(D,H)** of transverse sections of *Arabidopsis* Col-0 **(A–D)** and *ntt* **(E–H)** gynoecia. White arrows mark vascular tissues, which are lignified; red arrows mark the transmitting tract, which is not lignified, also delimited by the two white lines **(C,D,G,H)**; the gynoecium border is marked with dashed lines **(C,D,G,H)**. Scale bars represent 50 μm **(A–H)**.

In order to know whether all the signal detected with Toluidine blue and Phloroglucinol (the darker staining) in the medial region corresponds to lignin, we performed an acriflavine staining and as well we analyzed the UV-autofluorescence signal in gynoecia cross-sections, both as indications of lignin presence (Donaldson et al., 2001; Bonawitz et al., 2014). We detected similar patterns in the vasculature tissues (white arrows, **Figures 3A–D**), indicating that this is indeed lignin. However, the darker staining observed in the transmitting tract with Toluidine blue and Phloroglucinol (red arrows, **Figures 3A–D**) was not detected with the acriflavine stain nor with UV-autofluorescence. This indicates that the compound(s) present in the transmitting tract is not lignin. However, the apparent compound(s) seems to be related to the transmitting tract, because the darker staining observed with Phloroglucinol and Toluidine blue is not visible anymore in the mutant for *NO TRANSMITTING TRACT* (*NTT*), which lacks a transmitting tract (**Figures 3E,F**; Crawford et al., 2007).

In conclusion, the signal observed with Toluidine blue and Phloroglucinol staining coincides with the signal observed with Alcian blue staining of acidic compounds in the transmitting tract; however, these signals do not reflect lignin presence. But interestingly, Toluidine blue and Phloroglucinol staining marks where the transmitting tract will be formed before it is detected by Alcian blue.

### Cell Wall Remodeling During Transmitting Tract Formation

Transmitting tract cells produce an ECM containing glycoproteins, glycolipids, and polysaccharides that facilitates pollen tube growth (Lennon et al., 1998; Crawford and Yanofsky, 2008).

The Alcian blue staining has been commonly used for the staining of acidic mucosubstances: proteoglycans and glycoproteins (Barrett, 1971; Kiernan, 2010). This dye has different staining properties according to the pH of the staining solution. The most widely used is at pH 3.1 (**Figure 2**), but in order to detect other compounds we performed the staining at (1) pH 1.0, for staining only sulphated glycosaminoglycans and glycoproteins and at (2) pH 2.5, for hyaluronic acid and all other acid glycoproteins (Tolivia and Tolivia, 1987; Kiernan, 2010). Although the intensity of the staining was different at different pH levels, the staining pattern was similar, suggesting the presence of sulphated polysaccharides (Supplementary Figures 2A,D) and hyaluronic acid and/or glycoproteins in the medial region (Supplementary Figures 2B,E). In this experiment, we left out the counter stain Neutral red as it could mask the blue stain (**Figure 2**).

In conclusion, we detected similar patterns between Alcian blue staining at all three pH levels (**Figure 2** and Supplementary Figure 2), and with Phloroglucinol (Supplementary Figure 2).

### Acriflavine Staining as a Marker of Ovule Lineage

**FIGURE 4:**
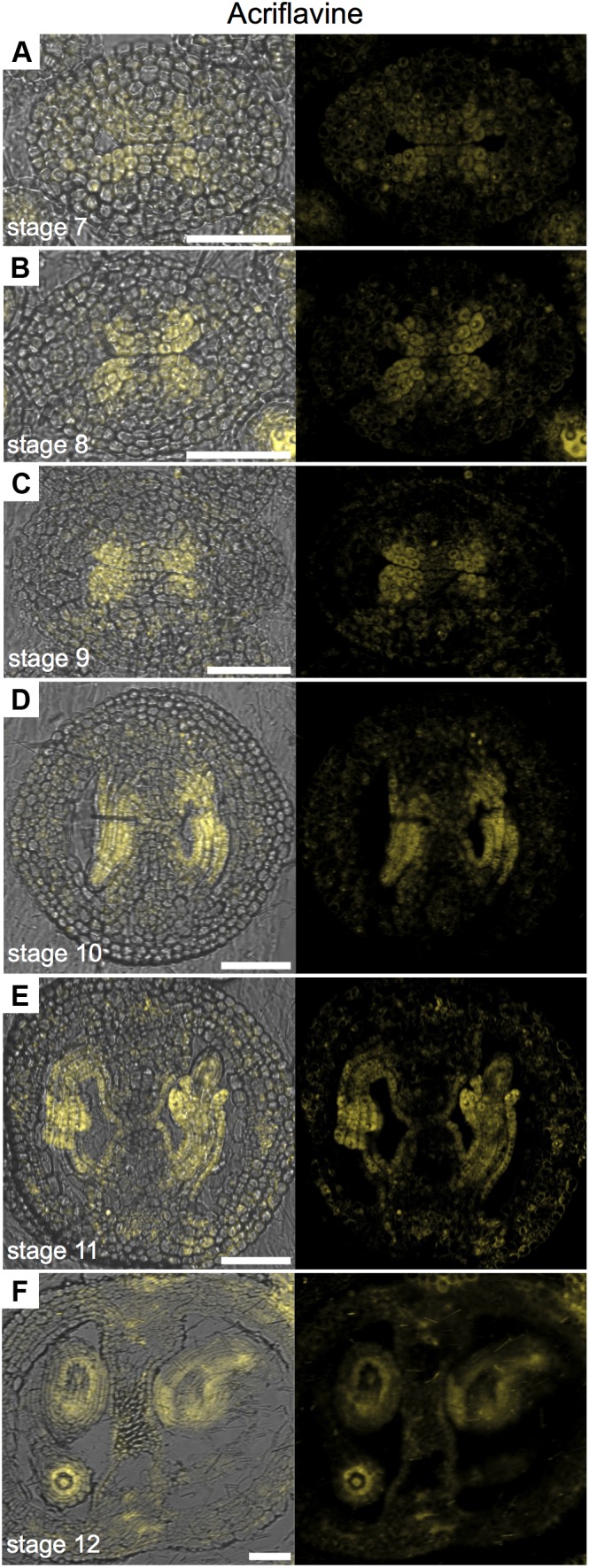
Acriflavine staining of transverse sections of *Arabidopsis* gynoecia. **(A–F)** Fluorescence signal of acriflavine for non-lignified tissue in transverse sections of *Arabidopsis* gynoecia at stages 7 **(A)**, 8 **(B)**, 9 **(C)**, 10 **(D)**, 11 **(E)**, and 12 **(F)**. Acriflavine signal was detected using a LP 575-nm filter. Scale bars represent 50 μm **(A–F)**.

In order to generate more data on lignin deposition, we used Acriflavine, a fluorescent dye that has been used in studies on lignin distribution (Donaldson et al., 2001; Bonawitz et al., 2014). Using this dye, signal from lignified tissue can be detected at 530 nm and non-lignified tissue at 600 nm (Donaldson et al., 2001). We observed similar patterns using the Phloroglucinol, Toluidine blue, UV-autofluorescence, and Acriflavine in detecting lignified tissues (Supplementary Figure 3), as observed by others (e.g., Hossain et al., 2012; Bonawitz et al., 2014; Liu et al., 2015). When we observed the emission signal for non-lignified tissue with a CLSM, we found a very interesting pattern in the medial region (**Figure 4**). The signal was detected in cells belonging to the ovule lineage. Signal was detected at stages 7 and 8 in the CMM, presumably mainly in the placentae (**Figures 4A,B**) and at stages 9 and 10 it preferentially localized in ovule primordia (**Figures 4C,D**). During ovule development, the signal maintained clearly detectable in, e.g., integuments, nucellus, as well as in the funiculi (**Figures 4E–G**). Note that some signal can also be found in the septum epidermis and valves (**Figures 4E,F**).

In conclusion, specially observing the fluorescence signal for non-lignified tissue permits the use of Acriflavine as a marker for the placenta and the ovule lineage.

### Cellulose Is Uniformly Distributed

**FIGURE 5:**
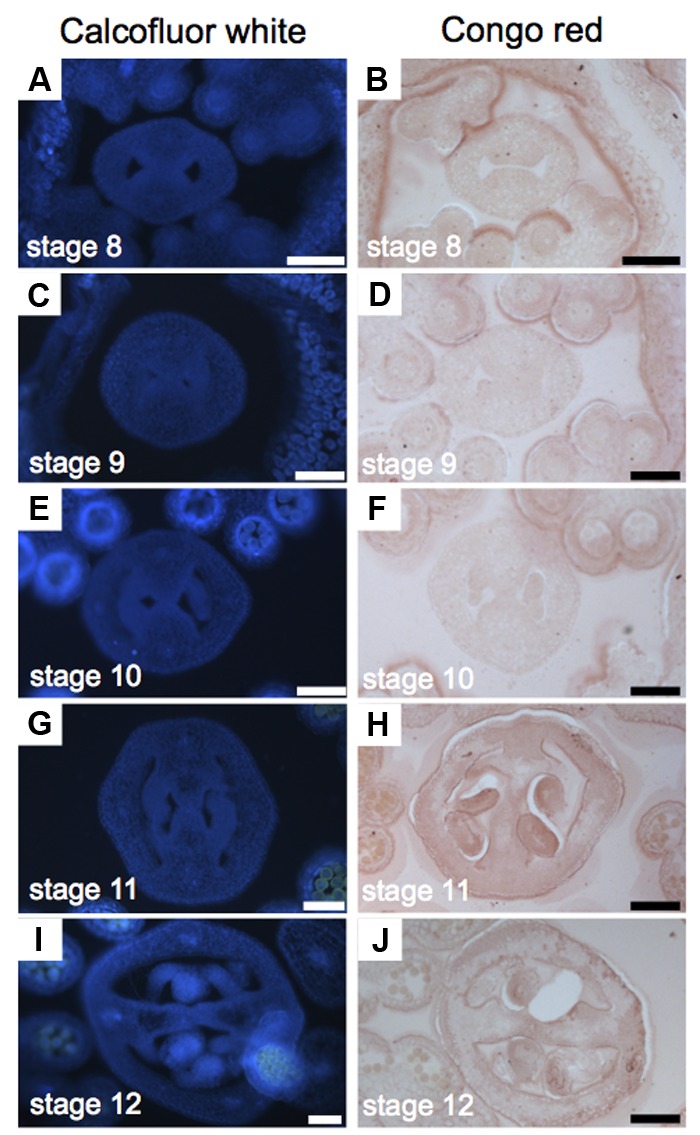
Cellulose distribution in transverse sections of *Arabidopsis* gynoecia. **(A–J)** Transverse sections of *Arabidopsis* gynoecia stained with Calcofluor white **(A,C,E,G,I)** or Congo red **(B,D,F,H,J)** at stages 8 **(A,B)**, 9 **(C,D)**, 10 **(E,F)**, 11 **(G,H)**, and 12 **(I,J)**. Scale bars represent 50 μm **(A–J)**.

Cellulose is a polysaccharide of glucose units and makes up most of the cell walls of plants (Cosgrove, 2005). For the study of cellulose distribution, we used Congo red and Calcofluor white, its fluorescent equivalent, obtaining similar results (**Figure 5**). Calcofluor white stains cellulose, callose, and other β-glucans (Maeda and Ishida, 1967; Hughes and Mccully, 1975; Wood, 1980; Mori and Bellani, 1996), while Congo red typically binds to β-1-4-glucans and cellulose (Wood, 1980). The presence of cellulose is, in general, uniform during the gynoecium formation (**Figure 5**). A stronger signal was detected in petals and stamens (**Figures 5A–F**), and also in the developing ovules (**Figures 5G–I**).

### Mannan Distribution Decreased Through Medial Domain Development

**FIGURE 6:**
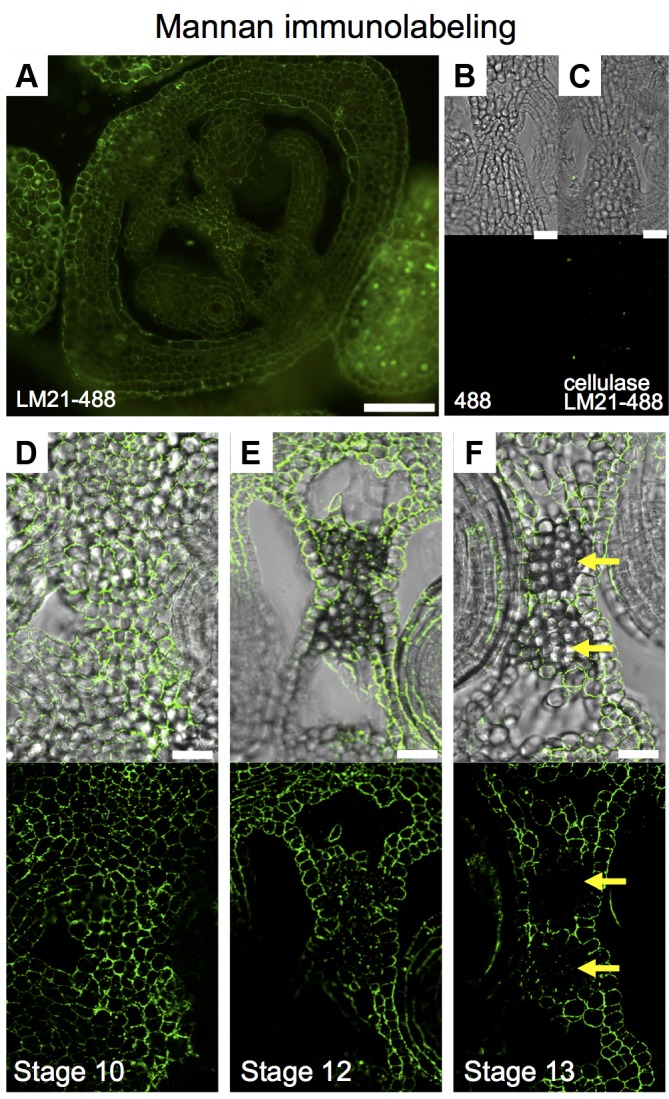
Mannan polysaccharide distribution in the *Arabidopsis gynoecium*. **(A,D–F)** Immunofluorescence signal of the LM21 anti-mannan antibody coupled to DyLight 488 (secondary antibody) in transverse sections of *Arabidopsis* gynoecia. Mannan polysaccharide distribution in the septum at stages 10 **(D)**, 12 **(E)**, and 13 **(F)**. **(B,C)** Negative controls; fluorescence signal is not detected in sections incubated with only the secondary antibody **(B)** or in samples pre-treated with cellulases **(C)**. Scale bars represent 50 μm in **(A)** and 20 μm in **(B–F)**. Yellow arrows mark transmitting tract cells that have no signal anymore, indicating cell wall degradation.

We wanted to implement immunolabeling followed by fluorescence microscopy analysis in gynoecia sections. Based on unpublished data, we chose for the detection of mannans; however, the protocol is not limited to the detection of other compounds with other available antibodies. Mannans are one of the most predominant polysaccharides in secondary plant cell walls, having storage and structural functions (Moreira and Filho, 2008; Schroder et al., 2009; Marcus et al., 2010). For the study of mannan distribution, we performed immunolocalization using the LM21 antibody, part of a set of monoclonal antibodies directed against plant cell wall components, which binds effectively to β-(1→4)-manno-oligosaccharides; in general, it displays a wide recognition of mannan, glucomannan, and galactomannan polysaccharides (Ordaz-Ortiz et al., 2009; Marcus et al., 2010). Using a secondary fluorescent antibody, we detected a clear and localized fluorescence signal, indicating the indirect detection of mannan in cell walls in the ovary of the gynoecium (**Figure 6A**). As negative controls, we used samples previously treated with macerozyme and cellulase (complete degradation of cell walls), or without the addition of primary antibody, resulting in almost a complete lack of signal in both cases (**Figures 6B,C**). After setting up the immunolocalization, we focused on the medial domain of the ovary. Strong anti-mannan signal was located in the whole septum at early developmental stages (**Figure 6D**), and we detected decreased signal at later developmental stages, especially where the transmitting tract gets formed (**Figure 6E**). At stage 13, when the gynoecium is mature and fertilization occurs, almost no signal was detected in the transmitting tract tissue (yellow arrows, **Figure 6F**), indicating the degradation of cell walls.

### Neutral Lipid Redistribution Accompanies Septum Fusion

**FIGURE 7:**
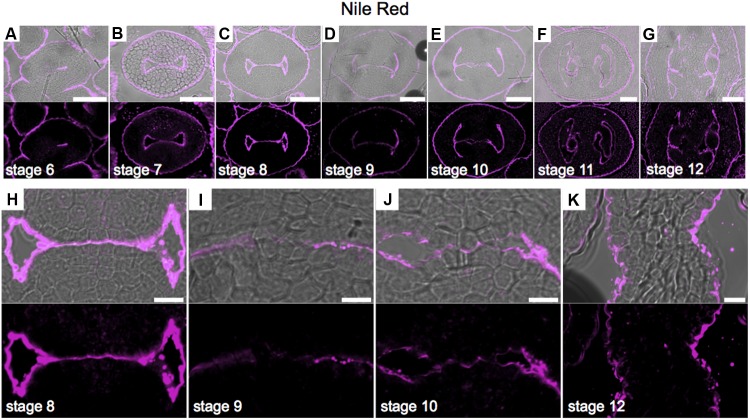
Neutral lipid distribution in transverse sections of *Arabidopsis* gynoecia. **(A–K)** Nile red staining of transverse sections of *Arabidopsis* gynoecia at stages 6 **(A)**, 7 **(B)**, 8 **(C)**, 9 **(D)**, 10 **(E)**, 11 **(F)**, and 12 **(G)**. Close-up of the *gynoecium* medial region where the two septa primordia will fuse, at stages 8 **(H)**, 9 **(I)**, 10 **(J)**, and 12 **(K)**. Note, also other floral organs have signal in the epidermis. Scale bars represent 50 μm **(A–G)** and 10 μm **(H–K)**.

Lipids have an important role in several biological processes (Chapman et al., 2012). To study lipid distribution in the medial domain cells, we used Nile red that stains lipid droplets composed of neutral lipids and triacylglycerols (Chapman et al., 2012; Kuntam et al., 2015). We detected a strong signal in the abaxial and adaxial carpel epidermis (and/or cuticle) during all stages of development (**Figures 7A–G**). A clear signal was detected in epidermis inside the young gynoecium before septum fusion (**Figure 7H**). Once the two septa primordia started to fuse, this signal decreased in those cells directly involved in the fusion process (**Figures 7I,J**). At stage 12, only signal was observed in the epidermis (and/or cuticle) of the septum, and not anymore where the two septa primordia fused (**Figure 7K**).

## Discussion

**FIGURE 8:**
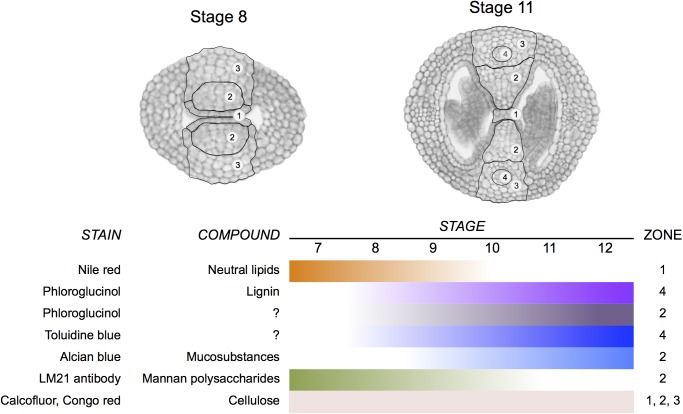
Summary of changes in cell wall composition during gynoecium medial domain development in *Arabidopsis*. The stains reveal the distribution of neutral lipids, lignin, mucosubstances, mannan polysaccharides, and cellulose in four zones during gynoecium development.

A correct formation of the gynoecium medial domain is crucial for *Arabidopsis* reproduction, since it contains the necessary tissues required for fertilization and subsequently seed formation (Reyes-Olalde et al., 2013). In this work, we detected changes in cell wall composition during medial domain development using different staining methods and immunolocalization. Two regions are particularly dynamic in cell wall composition modifications during development (**Figure 8**), one is the septa primordia, where epidermal cells have to prepare to fuse to form the septum and the second one is related to those changes occurring in the center of the septum for the formation of the transmitting tract, topics in detail described previously (Alvarez and Smyth, 2002; Crawford et al., 2007). Both processes, septum fusion and transmitting tract formation, have major effects on fertilization and therefore, on reproductive competence.

### Organ Fusion and Lipids

Organ fusion, including carpel fusion (or septum fusion), has always been an interesting and important research area (Verbeke, 1992; Tanaka and Machida, 2007). In the case of *Arabidopsis*, several mutations produce floral organ fusions (Lolle et al., 1998), some examples are mutations in *FIDDLEHEAD* or *HOTHEAD* (Lolle et al., 1998; Krolikowski et al., 2003). In many cases, defects in organ fusion or separation are the result of alterations in cuticle composition, present on the outside of the epidermal cell wall (Lolle et al., 1998; Krolikowski et al., 2003). The epidermal cuticle is a complex matrix of cutin, polysaccharide microfibrils, and waxes, serving as a selective barrier (Nawrath, 2006). Lipids are part of the cuticle and are important for, e.g., plant defense (Chapman et al., 2012), reproduction (Hsieh and Huang, 2007), and development (Yeats and Rose, 2013). The Nile red staining allowed us to track how lipids participate in, or at least coincide with, the process of septa primordia fusion.

Those genes reported as regulators of carpel and septa primordia fusion (e.g., Heisler et al., 2001; Alvarez and Smyth, 2002; Nahar et al., 2012; Kamiuchi et al., 2014; Vialette-Guiraud et al., 2015), or those expressed at early gynoecium formation (Reyes-Olalde et al., 2013), could be directly regulating the expression of genes that shape the cuticle properties or composition. This will be interesting to study in the future.

### Transmitting Tract Formation and Polysaccharides

A correct formation of the transmitting tract allows successful fertilization and reproduction (Alvarez and Smyth, 2002; Crawford et al., 2007). Mutations in genes such as *NTT* (Crawford et al., 2007), or in some bHLH transcription factors such as *SPATULA* (*SPT*; Alvarez and Smyth, 1999), *HECATE* (*HEC*; Gremski et al., 2007), or *HALF FILLED* (*HAF*) in combination with *BRASSINOSTEROID ENHANCED EXPRESSION 1* (*BEE1*) and *BEE3* (Crawford and Yanofsky, 2011) lead to altered transmitting tract formation.

An important progress has been achieved in the study of transmitting tract structure and composition (Lennon et al., 1998; Crawford and Yanofsky, 2008), and also a link with cell death programs controlled at genetic level has been proposed (Crawford et al., 2007). In this work, we observed some cell wall modification, but not lignification, preceding and accompanying the formation of the transmitting tract, this could be a starting point in the study of the control of this process at the biochemical level and suggest the involvement of a set of enzymes.

As mentioned above in the section on organ fusion and lipids, it will be interesting to investigate whether those genes reported as regulators of transmitting tract formation (Alvarez and Smyth, 1999; Crawford et al., 2007; Gremski et al., 2007; Crawford and Yanofsky, 2011), or those expressed at early gynoecium formation (Reyes-Olalde et al., 2013) are directly regulating the expression of genes that affect cell wall polysaccharide composition.

### The Use of Fluorescent Dyes in the Study of Gynoecium Development

Fluorescent dyes are an important tool in the study of cellular dynamics. During gynoecium development, we obtained similar results in the case of the detection of cellulose (Calcofluor white vs. Congo red), and in the case of the detection of lignin in the vasculature (Phloroglucinol, Toluidine blue, Acriflavine, and UV-autofluorescence) using different dyes or methods. The advantage of using fluorescent dyes is the good resolution of the histological images and the advantage of using confocal laser scanning microscopy, allowing detailed studies at the cellular level. For instance, PI allows excellent visualization of cells (Supplementary Figure 4). Especially interesting, and unexpected, we found that the fluorescent compound Acriflavine stains part of the CMM, placenta, funiculus, and the developing ovule; so, the complete ovule lineage. This pattern we observed when obtaining the emission spectrum at 600 nm (LP 575-nm), which is supposed to be specific for the staining of non-lignified tissues. In animals, Acriflavine was identified as an inhibitor molecule by binding to ARGONAUTE2 (AGO2; Madsen et al., 2014), as AGO proteins in plants, AGO2 is important for sRNAs functioning (Fagard et al., 2000; Bartel, 2004). Intriguingly, sRNAs have been shown to be important for the germline in plants (Olmedo-Monfil et al., 2010), and Acriflavine marks the female germline. We have no information at the moment what it biologically means, but at least it is making Acriflavine a very interesting marker for different studies. In our opinion, it is a good example of using less common dyes in developmental plant biology.

### Immunofluorescence Is Not Commonly Used in the Study of Gynoecium Development, but Offers Opportunities

A technique that maintains the tissue intact and also permits detailed cell wall analysis is immunofluorescence, i.e., immunolabeling followed by fluorescence microscopy analysis (e.g., Willats et al., 1999; Marcus et al., 2010). However, this technique is hardly used to study gynoecium development. We took advantage of one of the antibodies available against cell wall components. The detection of mannan polysaccharides has been reported in a wide number of cell types in *Arabidopsis* (e.g., Handford et al., 2003; Moller et al., 2007; Goubet et al., 2009; Marcus et al., 2010), though mostly in homogenized tissues. The antibody LM21 displays a wide recognition of mannan, glucomannan, and galactomannan polysaccharides (Marcus et al., 2010). The use of indirect immunolocalization is an important tool for the detection of modifications in specific cells. The protocol we set-up allows fast and easy detection of cell wall compounds in the gynoecium, and assumedly suitable as well for the detection of other compounds, metabolites, or proteins. It will be interesting to perform further detailed studies during gynoecium development in wild type and in mutant plants to better understand the importance of cell wall modifications.

## Conclusion

We identified several changes in cell wall composition and modifications during especially septum fusion and transmitting tract formation, summarized in **Figure 8**. With this information we can start deciphering what are the enzymes directly performing those activities and study if the master regulators of early gynoecium development control them.

## Author Contributions

HH-U and SdF conceived the project. HH-U performed the experiments. HH-U and SdF wrote and edited the manuscript. Both authors read and approved the final manuscript.

## Conflict of Interest Statement

The authors declare that the research was conducted in the absence of any commercial or financial relationships that could be construed as a potential conflict of interest.
