# Supplementary material for: Exploring Cell Wall Composition and Modifications During the Development of the Gynoecium Medial Domain in Arabidopsis
Source: Front Plant Sci. 2018 Apr 12;9:454. doi: 10.3389/fpls.2018.00454 (PMC5906702; doi:10.3389/fpls.2018.00454)
Supplement: Supplementary file 1 [file Image_1.PDF]

Supplementary Material

Exploring cell wall composition and modifications during the development of the gynoecium medial domain in Arabidopsis

Humberto Herrera-Ubaldo and Stefan de Folter*

*** Correspondence:** Stefan de Folter: stefan.defolter@cinvestav.mx

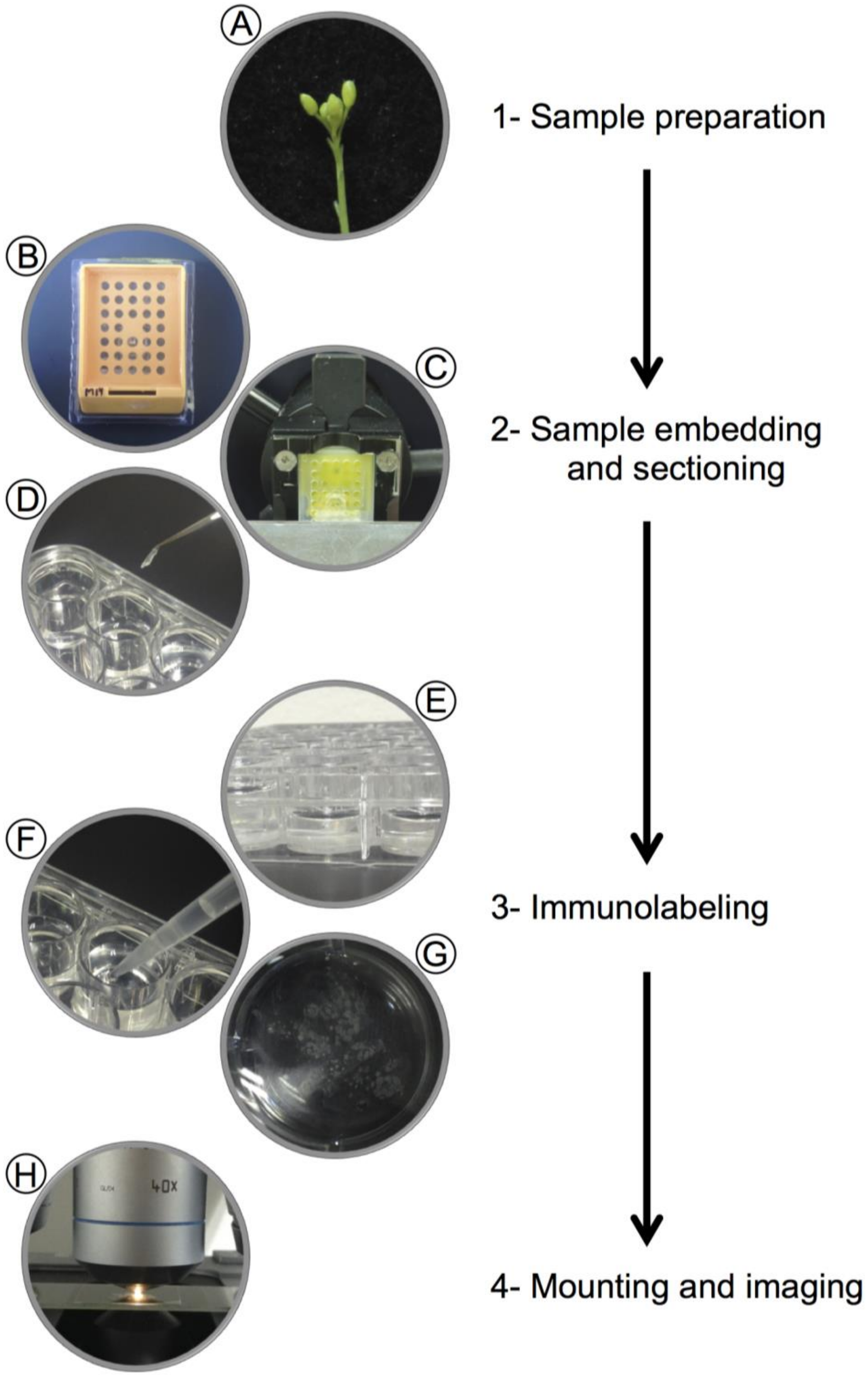

Supplementary Figure 1. General overview of the immunolabeling protocol. Arabidopsis inflorescences are collected **(A)** and embedded in a resin **(B)**, microtome sections **(C)** are transferred **(D)** to a 24-well plate **(E)** for the subsequent treatments and hybridization steps **(F)**, free-floating tissue sections can be observed inside the well **(G)**; after mounting, samples are observed in a fluorescence or confocal microscope **(H)**.

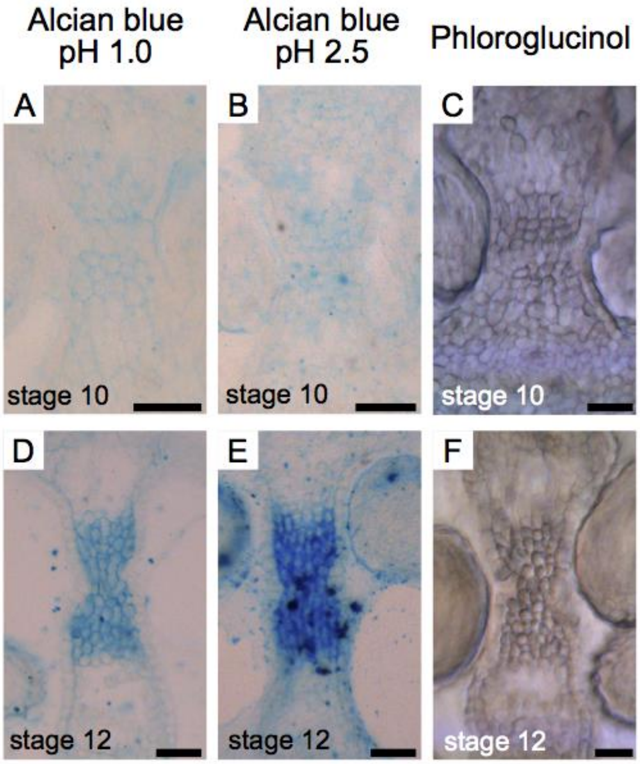

Supplementary Figure 2. Alcian blue and phloroglucinol staining of transverse sections of Arabidopsis gynoecia. Staining of the transmitting tract with alcian blue at pH 1 **(A,D)**. Staining of the transmitting tract with alcian blue at a pH 2.5 **(B-E)**. Staining of the transmitting tract with phloroglucinol **(C,F)**. Scale bars represent 25 μm **(A-F)**.

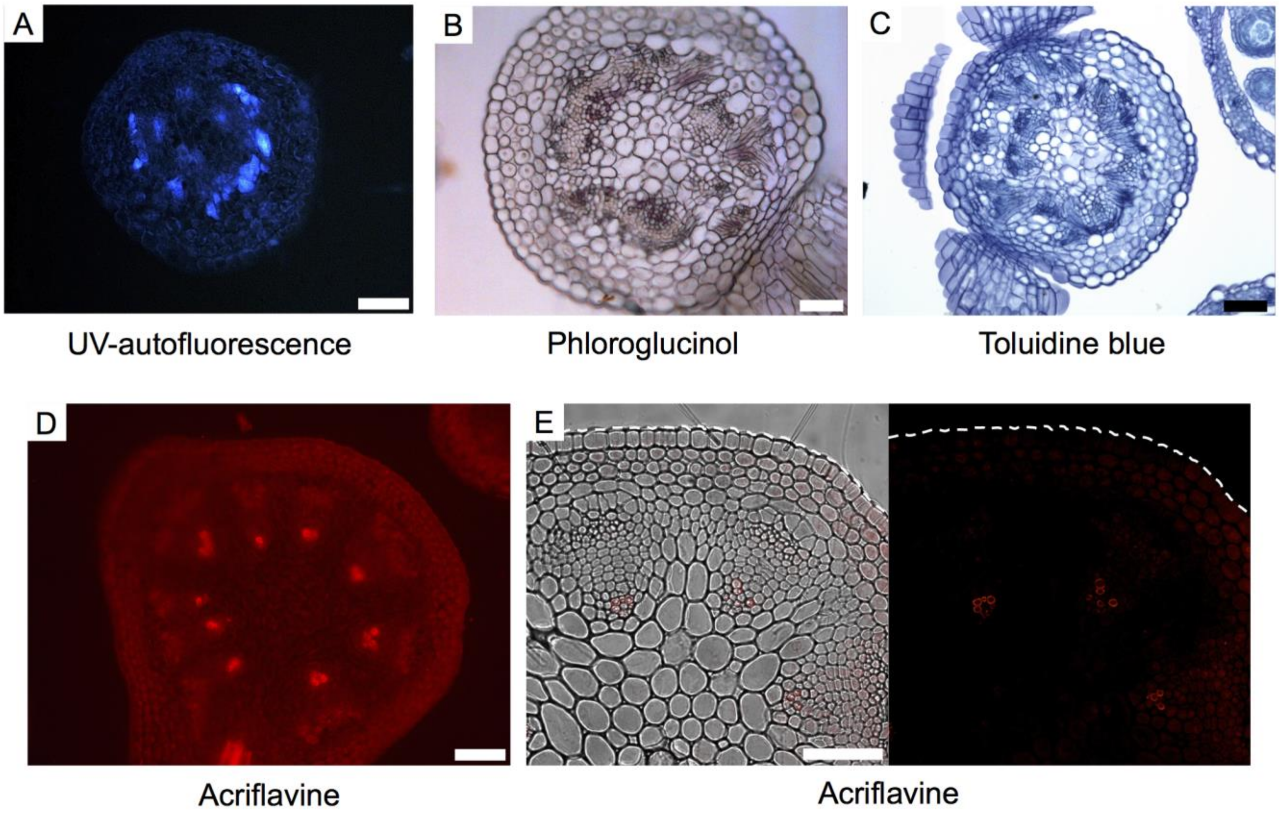

Supplementary Figure 3. Lignification patterns in inflorescence shoots. The UV-autofluorescence **(A)**, phloroglucinol **(B)**, toluidine blue **(C)**, and acriflavine staining **(D** and **E)** reveal similar lignification patterns in cross-sections of an inflorescence shoot. Acriflavine signal was detected at 575 nm in (**D)** and at 500-520 nm in **(E)**. Dashed lines in (**E)** mark the tissue border. Scale bars represent 50 µm **(A-E)**.

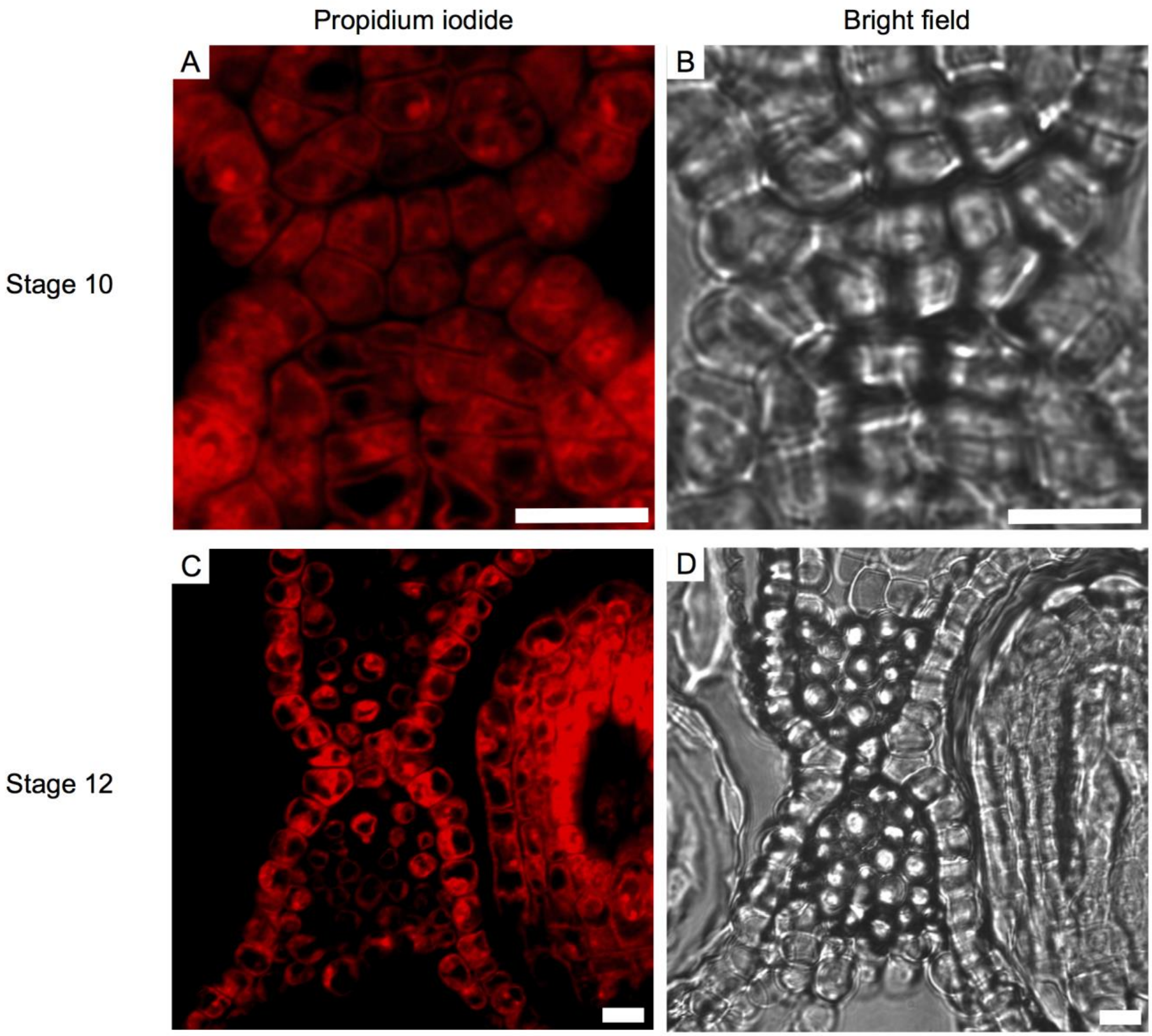

Supplementary Figure 4. Comparison between fluorescence and bright field microscopy in the study of gynoecium development. Propidium iodide staining of transverse sections of Arabidopsis gynoecia at stage 10 **(A)** and stage 12 **(C)**, and their bright field equivalent **(B** and **D)**. Scale bars represent 10 μm **(A-D)**.
